# Multifaceted Microcephaly-Related Gene MCPH1

**DOI:** 10.3390/cells11020275

**Published:** 2022-01-14

**Authors:** Martina Kristofova, Alessandro Ori, Zhao-Qi Wang

**Affiliations:** 1 Leibniz Institute on Aging—Fritz Lipmann Institute (FLI), Beutenbergstrasse 11, 07745 Jena, Germany; Martina.Kristofova@leibniz-fli.de (M.K.); Alessandro.Ori@leibniz-fli.de (A.O.); 2 Faculty of Biological Sciences, Friedrich-Schiller University of Jena, Bachstrasse 18k, 07743 Jena, Germany

**Keywords:** MCPH, neurogenesis, gonad development, tumorigenesis, mouse models

## Abstract

MCPH1, or BRIT1, is often mutated in human primary microcephaly type 1, a neurodevelopmental disorder characterized by a smaller brain size at birth, due to its dysfunction in regulating the proliferation and self-renewal of neuroprogenitor cells. In the last 20 years or so, genetic and cellular studies have identified MCPH1 as a multifaceted protein in various cellular functions, including DNA damage signaling and repair, the regulation of chromosome condensation, cell-cycle progression, centrosome activity and the metabolism. Yet, genetic and animal model studies have revealed an unpredicted essential function of MPCH1 in gonad development and tumorigenesis, although the underlying mechanism remains elusive. These studies have begun to shed light on the role of MPCH1 in controlling various pathobiological processes of the disorder. Here, we summarize the biological functions of MCPH1, and lessons learnt from cellular and mouse models of MCPH1.

## 1. Neurogenesis and Brain Size Determination

To ensure the development of the unusually large human brain to its size and complexity, highly precise mechanisms must govern temporal-spatial processes of the neuroprogenitor cells (NPCs) and their derivatives for their proliferation and differentiation. These processes have been extensively studied in the context of brain developmental disorders and in evolution. Brain size at birth is largely determined by the relative rate of proliferation and differentiation of NPCs during embryonic neurogenesis [1,2]. NPCs originate from the neuro-epithelium of the ventricular zone of the neural tube (dorsal telencephalon) [3,4]. During early neurogenesis, neuroepithelial cells initially proliferate to expand the progenitor pool, and later undergo differentiative divisions into daughter progenitors and neurons [5,6]. NPCs have an apical-basal polarity [4,7] and their nuclei have the ability to move along the apical-basal axis (interkinetic nuclear migration), which is coordinated tightly by the cell cycle; yet, the reasons behind this are still debatable and unclear [8,9,10]. It is well established that NPCs’ ability to proliferate has the main impact on brain size. Firstly, expansion of the neuroprogenitor pool is achieved via symmetric divisions ensuring their maintenance and self-renewal, to produce two identical cell types. On the other hand, asymmetric division produces one progenitor cell and one neuron. Finally, a specific type of symmetric division, i.e., symmetric differentiative division of NPCs, produces two daughter neurons.

Many factors influence which one of the division modes is favored and this process is tightly regulated. Among other factors, cell cycle length is one of the examples, with a longer G1 phase deciding for differentiation and a shorter one favoring self-renewal [11]. Interestingly, an experimentally shortened or prolonged G1 phase is sufficient to alter the division mode [12,13]. Another well-studied mechanism is related to the cell polarity. The relationship between the axis of cell polarity and the orientation of the cleavage plane (which is orthogonal to the mitotic spindle) is critical in deciding whether the cells undergo a symmetric or an asymmetric division [14,15]. According to this hypothesis, cell fate determinants are unequally distributed in the dividing parent cell. If the mitotic spindle orientation is parallel to the polar axis of the cell, the two daughter cells receive differential determinants and/or extracellular cues, resulting in an asymmetric division. On the other hand, when the polar axis and the mitotic spindle are in the perpendicular orientation, cells are more likely to divide symmetrically [16]. Therefore, the activity and function of the centrosome plays a critical role in these processes because the centrosome regulates spindle alignment, cilia formation, and the distribution of cell fate determinants during cell cycle progression [16]. In sum, the genetic and epigenetic factors that regulate the activity and function of all these cellular apparatuses are critical for neurogenesis and thereby determine brain size.

## 2. Genetics and Etiology of Microcephaly (MCPH)

Human primary microcephaly (MCPH, OMIM251200) is a neurodevelopmental disorder, characterized by a small brain at birth and clinically defined by a head circumference of more than three standard deviations below the mean compared to age-, sex-, and ethnicity-matched individuals [1,17]. The incidence of MCPH varies from 1:30,000 to 1:250,000 depending on the population and is generally high in populations in which consanguineous marriages are common [17,18]. Patients’ IQ values are between 30% and 80% of the average of normal individuals, depending on the severity of the microcephaly [17]. The gross reduction in brain size mainly affects the prefrontal cortex, which otherwise exhibits a normal brain architecture, with all cortical layers developed. This suggests reduced cell divisions during neurogenesis [19] rather than neuronal cell death.

To date (13 January 2022), 29 loci (Table 1) have been identified as being causal for MCPH, with 28 loci listed in the Online Mendelian Inheritance in Man (OMIM) database (https://omim.org/entry/251200, accessed on 11 January 2022) [20,21], and it is likely that this list will grow in the future thanks to advances in “omics” methods, which enable better identification of disease-causing variants. The continuous expansion of the list clearly demonstrates a high genetic heterogeneity of MCPH. Many of these genes have been implicated in various molecular and cellular processes, which contribute to the etiology of MCPHs. Strikingly, the vast majority of them play a role in centrosome function and mitotic spindle alignment [21,22]. The centrosome, as a major organizer of microtubules, is known to regulate the position and dynamics of the microtubule structure, such as the mitotic spindle alignment and cilia formation. Thus, primary microcephaly is often regarded as a form of *ciliopathy*, a group of diseases characterized by dysfunctional cilia [23,24,25]. During mammalian brain development, the centrosome controls the polarized cell behavior of neural cells from progenitor fate determination to neuronal migration and axonal growth navigation [24,25,26]. Hence, the maintenance of centrosome biogenesis, stability, and functionality is crucial for brain development, which nevertheless are very vulnerable to any pathological perturbations [23,27].

Many recent reviews have already discussed other MCPH genes [20,21,28]. In this short article, we will focus on the MCPH1 gene, a founding member of the MCPH family, and summarize the genetic and molecular function of MPCH1.

## 3. Primary Microcephaly Type 1—MCPH1

Primary microcephaly type 1, historically also referred to as *microcephalia vera*, is the first discovered MCPH gene and is also allelic to the premature chromosome condensation syndrome (PCC, OMIM606858), characterized by a high proportion of prophase-like cells [30,67]. Another autosomal recessive condition of microcephaly associated with craniofacial abnormalities [68] was later found to be caused by mutations in the same MCPH1 gene [69]. Some MCPH1 patients also exhibit growth retardation, a feature typical of many “genetic instability disorders” caused by mutations in DNA repair proteins, such as the Seckel syndrome (caused by mutations in Ataxia-Telangiectasia and Rad3-related (ATR)) and the Nijmegen breakage syndrome (NBS, caused by mutation in NBS1, *Nbn*) [19]. The cause of the microcephaly phenotype in these genetic instability syndromes is believed to be attributed to the proliferation defects and apoptosis of NPCs during embryonic development [70]. Therefore, initially, microcephaly or MCPH1 was extensively studied for its role in the DNA damage response (DDR), including DNA repair, cell cycle checkpoint activation, apoptosis, and transcription [19,71,72].

MCPH1 was the first locus identified as being responsible for MCPH1 [29,73] and is also one of the most commonly mutated genes, together with ASPM (MCPH5) and WDR62 (MCPH2) [20,28,74]. It is mapped to chromosome 8 [73] and the gene product was originally named Microcephalin [29]. It is also termed as BRIT1 (BRCT-repeat inhibitor of hTERT expression) because it was independently discovered in a genetic screen for regulators of the telomerase function [31], and later shown to be the same gene as MCPH1 [75]. Numerous MCPH1 mutations have been identified [76,77]. Notably, all missense mutations span the region of the N-terminal domain (exon 2 and 3), and result in non-conservative changes in amino acid residues. Amongst the most common patient mutations are nonsense 74C>G mutations leading to the premature stop codon Ser25X [17,29], or missense 80C>G mutation leading to a Tyr27Arg substitution [78]. Some of the frequently mutated amino acids are evolutionary conserved, for example, Thr27, which is conserved in mammals and amphibians [78], and Ser72 and Trp75, which are conserved in all vertebrates and Drosophila [79].

## 4. MCPH1 Protein Structure

The *MCPH1* gene is highly expressed in the human fetal brain, testis, liver, pancreas, and kidneys, yet at lower levels in other fetal and adult human tissues, such as the heart, lungs, thymus, and spleen [29,80]. In situ hybridization confirmed the expression in the developing forebrain in the region of the lateral ventricles, where NPC cells divide to produce neurons [81]. Expression of the *Mcph1* gene in mice slightly declines after birth [81]. The MCPH1 protein comprises 14 exons (in both mouse and human), which span across 835 amino acids (aa) and 200 kb of genomic DNA. The MCPH1 protein exists in two major isoforms: the full-length MCPH1 (MCPH1-FL; 835aa) and the second one is expressed from an alternative transcript lacking the last six exons (MCPH1Δe9–14; 611aa). Both isoforms are expressed in fetal tissues at a similar level and have no cell type specificity [82]. Interestingly, both isoforms are regulated antagonistically during the cell cycle: MCPH1-FL mRNA decreases from the mid-S phase to the G2 phase, while the transcript level of MCPH1Δe9–14 increases during this period [82]. Additionally, both isoforms act differently in DDR, where only MCPH1-FL associates with ionizing radiation (IR)-induced foci (IRIF) of γH2AX.

Structurally, MCPH1 contains three BRCT (BRCA C-terminal) domains, which were first described in BRCA proteins but can be found in many DDR proteins. Generally, these domains are 85-95 aa long with several conserved residues and often appear in repeats separated by variable linker regions no longer than 24 aa [83]. The structure of the BRCT domain is well characterized [83,84], where a central β-sheet is surrounded by 2 or 3 α-helices [85]. It is a highly conserved structure since these domains are found besides *Eukaryota* in another two super kingdoms *Archea* and *Bacteria* [86]. The BRCT domain mediates protein–protein interactions, often specifically BRCT–BRCT interactions [87]. The BRCT repeats preferentially interact with phosphorylated proteins, for example, in the DDR cascade [84,88]. These motifs can also recognize ADP-ribosylation [89], which is important for DDR signaling. The first BRCT1 domain (2-83 aa) lies within the N-terminal region and the tandem of BRCT2 and BRCT3 spans the C-terminus (320-399 aa and 432-502 aa, respectively) of MCPH1. The tandem of BRCT2 and BRCT3 directs self-oligomerization and is crucial for IRIF formation [32,90]. Several studies reported that MCPH1 interaction with its partners is mediated by BRCT domains [76]. Intriguingly, it is the N-terminal BRCT domain that is decisive in MCPH1-associated phenotypes, including brain size [91,92]. A poorly conserved Condensin II-binding motif is located in the middle region. Although three distinct NLS were identified in the MCPH1 sequence, one in the N-terminus, one in exon 8, and a third one in the BRCT3 in the C-terminus, the C-terminal NLS is responsible for nuclear localization [82]. In the middle domain, D-box (amino acids 752-755) and KEN-box (amino acids 599-601) degron (a short motif for degradation) sequences are found, which are recognized by the APC/C complex and mediate the degradation of MCPH1 in the G1 phase [93].

## 5. Cellular Function of MCPH1

### 5.1. MCPH1 Subcellular Localization

Co-localization of MCPH1 with γ-tubulin in U2OS cells suggests its putative centrosomal localization [94]. Similarly, ectopically expressed MCPH1 was found in the centrosome of human U2OS and chicken DT40 cells [32,95], which was attributed to its first BRCT domain in chicken cells [32], although other studies did not confirm the finding [90,96]. Indirect evidence of centrosomal localization was also obtained by experiments showing that the loss of MCPH1 leads to centrosome amplification and defective spindle assembly [97,98]. In response to DNA damage, MCPH1 forms IRIF and co-localizes with γH2AX in the nucleus [32]. Interestingly, immunofluorescence and electron microscopy of gold immunostaining revealed that MCPH1 is also associated with mitochondria in mouse and human NPCs [33].

### 5.2. Cellular Toxicity

It is known that the protein levels of MCPH1 are tightly regulated and prolonged overexpression of MCPH1 seems to be toxic to the cell [96,99], although the reasons are unclear. Injecting Xenopus embryos with *Mcph1* mRNA led to cell cycle arrest [96]. Some studies suggest a direct role of MCPH1 in apoptosis. For instance, MCPH1 overexpression in A549 lung carcinoma cells repressed uncontrolled cell proliferation via cell cycle arrest in the S and G2/M phases [100]. In these cancer cells, MCPH1 overexpression promoted cell apoptosis due to an increase in the Bax and active caspase-3 protein levels, as well as a decrease in the level of Bcl-2 [100].

### 5.3. MCPH1 and DNA Repair

An aberrant DDR is characteristic of MCPH1 patient cells. MCPH1 is a surveillance protein that acts on DNA damage sites by recruiting sensor and mediator proteins [75,101]. In this regard, it facilitates homologous recombination repair (HRR) of double-strand breaks (DSBs) [90,102]. MCPH1 also recruits the BRCA2 and RAD51 complex at IRIF, presumably DSB sites, via a direct interaction between the C-terminus of MCPH1 and the N-terminal domain of BRCA2 [103]. Upon genotoxic stress and at the IRIF site, MCPH1 co-localizes with many proteins, including γH2AX, mediator of DNA-damage checkpoint 1 (MDC1), p53-binding protein 1 (53BP1), ATM, ATR, CHK1, p-RAD17, and replication protein A (RPA34) [101,104,105]. Moreover, in response to UV radiation, the phosphorylation of RAD17 and RPA34, downstream effectors of the ATR pathway, is reduced in MCPH1-deficient cells [104,105]. Under replicative stress, MCPH1 recruits TopBP1 and thereby facilitates ATR activation upon its phosphorylation at S322 by ATM or ATR [99].

In addition, the N-terminal domain of MCPH1 can interact with the BAF170 subunit of the chromatin remodeling SWI/SNF complex to relax chromatin to allow DDR proteins to access DNA damage sites [80,106]. Its interaction with this complex is increased upon DNA damage in an ATR-dependent manner [106]. MCPH1 is associated with TRF2 at the telomere region [107] and this interaction allows homology-directed DNA repair and also promotes telomere replication during the S phase [108]. Consistently, MCPH1-deficient cells also show long telomeres, which is consistent with its telomerase-inhibiting role [109]. It was shown that MCPH1 is able to interact with the transcription factor E2F1 and is also involved in the transcriptional regulation of CHK1 and BRCA1, important proteins regulating cell cycle checkpoints in response to DNA damage [110]. Taken together, these studies demonstrate the important role of MCPH1 in regulating the DNA repair mechanisms and maintaining the genome integrity.

### 5.4. MCPH1 and Cell Cycle Control

MCPH1 has been described to regulate the activity of the cell cycle regulator CDC25A in an ATR-CHK1-dependent manner [71] and MCPH1 patient cells show a dysfunctional G2-M checkpoint mainly due to impaired degradation of CDC25A, causing premature entry into mitosis [71]. MCPH1 is also suggested to positively regulate some of the cell cycle checkpoint proteins, such as BRCA1 and CHK1, both being important for the G2/M transition [75,111]. MCPH1 via its centrosomal co-localization with CHK1 can directly interact with pericentrin [95]. The molecular mechanism by which MCPH1 regulates cell cycle progression was further demonstrated by its role in interacting with two E3 ubiquitin ligases: together with the βTrCP2 subunit of the SCF complex it regulates the S-phase progression and G2-M transition [112], while it interacts with two subunits of APC/C, CDC27, and CDH1 in late mitosis in order to secure the entry of the G1 phase [112,113]. MCPH1 competes with Condensin II on chromatin [114] and the lack of MCPH1 promotes the binding of Condensin II to chromatin before all necessary mitotic structures are formed by the end of the G2 phase, ultimately leading to the PCC phenotype [30,115]. Hence, MCPH1 regulates the timely onset of mitosis, and it must be tightly regulated for degradation at the end of mitosis.

### 5.5. Emerging Role of MCPH1 in Metabolism

**Figure 1:**
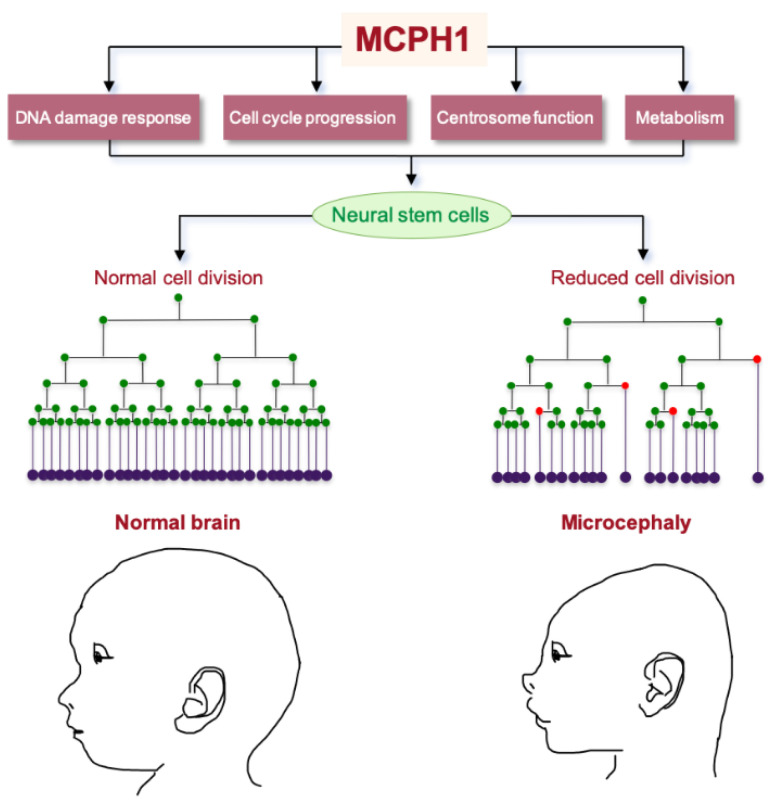
MCPH1 regulates brain size. MCPH1 is employed in many cellular functions that regulate the division mode of neural stem cells during embryonic brain development. Impairment of MCPH1 functions leads to an insufficient production of neurons (depicted in the diagram by dark purple dots) due to the premature differentiation of neural stem cells (depicted by red dots) and a reduction of self-renewal divisions (depicted by green dots). As a result, microcephaly manifests in affected individuals.

Recently, a role for MCPH1 in the regulation of mitochondrial activity and metabolism was reported [33], where MCPH1 directly interacts with VDAC1 and GRP75 in mouse and human NPCs to maintain glutaminolysis, a pathway involved in replenishing the tricarboxylic acid cycle and is vital for cell proliferation and survival [116,117]. Independently, in the proteomic screening of lipocalin-2 (LCN2)-deficient cells, which exhibited an altered mitochondrial function and fatty liver disease, MCPH1 was identified as a strongly downregulated protein [118]; however, it is unclear if this observation relates to its possible function at mitochondria or is a result of tumorigenesis in these mice. Further studies are required to assess the role of MCPH1 in the metabolic functions and its importance for NPCs’ maintenance (Figure 1).

## 6. Animal Models of MCPH1

MCPH1 is implicated in a wide range of cellular processes as discussed above. However, the exact molecular mechanisms of how it regulates the expansion of the neuroprogenitor pool and thereby brain size still remains poorly understood. To better understand the role of MCPH1 in neurogenesis, several mouse models have been generated (Table 2). While some have mostly faithfully mimicked the brain and cellular phenotypes observed in human patients and patient cells, others have only recapitulated some phenotypes. In this regard, it is worth noting that MCPH1 has multiple structural domains, and each engages in different functions in vivo. It should also be mentioned that the mouse ortholog of MCPH1 shares only 57% sequence homology with the human protein [29]. Below, we summarize all these studies.

### 6.1. Mcph1^−/−^ Mice

The first *Mcph1*-null mouse model was generated via a Cre/LoxP-targeted deletion of exon 2 and showed no obvious microcephaly phenotype [105]. However, these mice were smaller, with a body weight of only 80% of their wild-type littermates. Unexpectedly, these mice exhibited infertility of both males and females accompanied by a reduction in the size of the gonads. Both *Mcph1*^−/−^ mice and mouse embryonic fibroblasts (MEFs) isolated from them were hypersensitive to IR and had impaired mitotic and meiotic DDR signaling.

### 6.2. Mcph1^gt/gt^ Mice

Another mouse model was generated by a gene trap approach, where the LacZ vector was inserted between exons 12 and 13, leading to a deletion of most of the C-terminal BRCT domain [111]. This mouse model is hypomorphic, since it still contained a residual MCPH1 protein and mutant mice had a normal brain and body weight, unaltered DDR, and were fertile. Interestingly, the mutant mice had significantly shorter lives than their littermate controls, and the mouse fibroblasts displayed a pronounced PCC phenotype. The PCC phenotype of *Mcph1^gt/gt^* mice that lack the C-terminal BRCT3 domain raises the point that perhaps the BRCT domain’s ability to bind to phosphorylated proteins is implicated in the process of chromosome condensation, which is also accompanied by phosphorylated histones [119].

### 6.3. Mcph1-del Mice

The third and perhaps the most studied *Mcph1* mutant mouse model was generated by a Cre/LoxP-targeted deletion of exon 4-5 [81]. *Mcph1-del* mutant mice had a smaller brain at birth, even already visible at E13.5, and showed growth retardation after birth [81]. The reduction in the brain size manifests predominantly in the cortical area while the cerebellum was not affected [120]. These mice also showed infertility and a reduction in the gonad size of the testes and ovaries [91]. The mutant mice exhibited an impaired DDR as judged by a hypersensitivity to IR [120]. In the NPCs derived from these mice, the localization of Chk1 to centrosomes is abrogated, causing premature Cdk1 activation and premature mitotic entry, which uncouples mitosis from the centrosome cycle [81]. By analyzing NPCs’ cell cycle progression, a misalignment of chromosomes, lagging chromosomes, and deficient spindle checkpoint (multiple spindle poles) were observed in *Mcph1-del* mice [81]. As a result of the premature Cdk1 activation and the subsequent inadequate Cdc25a degradation, a shorter G2 phase was also reported, which leads to premature mitotic entry of NPCs [81]. Altogether, these molecular defects led to a premature switch from the symmetric to the asymmetric division mode and ultimately the exhaustion of the NPCs pool. Interestingly, overexpression of Chk1 in *Mcph1-del* mice failed to correct the microcephaly phenotype [77]. Notably, the expression of Chk1 is unaffected in human and murine MCPH1 cells [81,121]. It seems that Mcph1 does not regulate the expression of Chk1 but is directly or indirectly implicated in the regulation of its proper localization during the cell cycle. Moreover, Mcph1 also regulates the stability of Cdc25a by cooperation with E3 ligase βTrCP2 in order to ensure proper timing of mitotic entry [112]. These observations strongly suggest that additional pathways are affected by the MCPH1 deletion.

### 6.4. Mcph1^tm1a/tm1a^ Mice

Another hypomorphic *Mcph1*-deficient mouse model was generated by targeted deletion of exon 4 [122]. Mutant mice were infertile and had a reduced brain weight but without growth retardation. The defects in DNA damage repair were mild and only revealed by the increased prevalence of micronucleated normochromatic erythrocytes. Interestingly, this model showed an eye abnormality and hearing loss, which have not been reported in MCPH1 patients nor in other Mcph1-deficient mouse models.

### 6.5. Mcph1^lox/lox^; Emx1^kiCre/+^

In this conditional mouse model, *Mcph1* was inactivated specifically in the neocortical progenitors in the lateral neocortex of E12.5 embryos [33]. These mice developed microcephaly due to the mitotic anomalies associated with increased apoptosis in the neocortex. Mutant NPCs harbored fragmented mitochondria and decreased the ATPase activity. The authors demonstrated a direct interaction of Mcph1 with VDAC1 in NPCs, which stimulates glutaminolysis via the ATF4/PCK2 pathway. This study, for the first time, links Mcph1 with the metabolic pathways to secure the energetic supply for highly proliferating NPCs.

### 6.6. Mcph1-ΔBR1 Mice

With the notion of the fact that mutations in human MCPH1 patients are mostly in the N-terminal domain of Mcph1, *Mcph1-ΔBR1* mice were generated by a targeted deletion of the N-terminal BRCT1 domain [91]. Remarkably, these mutant mice sufficiently recapitulate all core phenotypes of complete knockout mice, such as microcephaly, infertility, PCC, and impaired DDR [81,105,120]. These observations strongly indicate the important role of the N-terminal BRCT domain in the etiology of MCPH1 and the brain size determination [91,92]. However, open questions remain as to how the N-terminal BRCT would influence brain development: (1) what is the contribution of other domains of the protein, and (2) what are those to-be-identified BRCT1–protein interaction partners, which would make the final decision regarding brain size.

Strikingly, infertility has been observed in several Mcph1-deficient mouse models, which is accompanied by atrophy of gonads, i.e., testes and ovaries, as well as tumorigenesis in these organs [81,91,105]. Thus, it is plausible that MCPH1′s role in maintaining a big brain and gonad development is important for positive selection in the primate lineage during evolution [123,124]. The high tumor penetrance in *Mcph1* mutant mice is also interesting, which is consistent with the observation that MCPH1 was found to be mutated in many malignancies, including breast, ovarian, and prostate cancer [125,126]. Many functions of MCPH1 can be attributed to tumorigenesis, for example, MCPH1′s role in DDR and cell cycle checkpoint activation, as well as in the maintenance of genome integrity. MCPH1 is also responsible for centrosome amplification [98], which is a common hallmark of many cancers.

Despite consistent phenotypes of impaired gonad development, infertility, and tumorigenesis in various MCPH1 mouse models [91,105,122], MPCH1 patients have not yet been reported for these symptoms. Some of the *Mcph1*-deficient mice have been reported to have a short lifespan [111,127], whereas other models did not display a short lifespan [81,91,122]. There is no systematic report on lifespan in MCPH1 patients, but one study reports MCPH1 patients in their 70 s [74]. In sum, the mouse model studies demonstrate a multifaced function of MCPH1 in various pathophysiological processes, even beyond brain development.

Finally, it is interesting to mention that transgenic rhesus monkeys overexpressing the human copy of the MCPH1 gene (*huMcph1-Tg monkeys*) showed a relative greater brain volume compared to non-transgenic control monkeys during early postnatal development [128]. Interestingly, these transgenic monkeys seem to have improved cognitive functions, i.e., short-term memory, although they had delayed neuronal maturation and differentiation [128].

## 7. Perspectives

MCPH1 plays pleiotropic roles in DDR, cell cycle progression and centrosome function, spindle alignment, chromatin remodeling, and metabolism. The *MCPH1* gene is ubiquitously expressed in tissues but, strikingly, only a limited number of cellular compartments, i.e., neuro-stem cells, and perhaps also primary germ cells, are particularly vulnerable to the mutation or loss-of-function of MCPH1. This high tissue specificity remains mysterious. It is possible that these tissue-specific stem cells are not equipped with alternative pathways to cope with any perturbations of the mechanisms elicited by MCPH1 loss. Or in parallel to this hypothesis, different functional domains of the MCPH1 protein can engage with different partners in a temporal-spatial manner under physiological and pathological conditions. Therefore, searching for partners that interact with different domains would be beneficial for understanding the specific as well as diverse functions of MCPH1. Finally, the dissection and understanding of the causal mutations in human *MCPH1* using more robust and feasible model systems, which even closely reassemble human patients, such as human or primate brain organoids, may provide insight into the etiology of microcephaly and cognitive disorders.

## Figures and Tables

**Table 1:** Genes associated with human MCPH (January 2022).

Disease ID	Chromosome Location	Inheritance	Gene	Protein	Subcellular Location	Cellular Function	Reference
**MCPH1**	8p23.1	AR	MCPH1	Microcephalin 1/ BRIT1	Nucleus/Centrosome/ Mitochondria	DNA damage response, chromatin condensation, cell cycle control	[29,30,31,32,33]
**MCPH2**	19q13.12	AR	WDR62	WD Repeat-containing protein 62	Centrosome/Spindle poles	Centriole biogenesis, spindle assembly	[34,35,36,37]
**MCPH3**	9q33.2	AR	CDK5RAP2	CDK5 regulatory subunit-associated protein 2	Nucleus/ Centrosome	Centriole biogenesis, spindle checkpoint, cytokinesis	[38,39]
**MCPH4**	15q15.1	AR	CASC5 (KNL1)	Cancer susceptibility candidate 5/Kinetochore scaffold 1	Kinetochore	Kinetochore attachment Spindle checkpoint	[40]
**MCPH5**	1q31.3	AR	ASPM	Abnormal spindle microtubule assembly	Nucleus/ Centrosome/ Midbody	Centriole biogenesis, spindle assembly, cytokinesis	[35,41]
**MCPH6**	13q12.12-q12.13	AR	CENPJ (SAS-4, CPAP)	Centromere protein J,	Centrosome	Centriole biogenesis	[38]
**MCPH7**	1p33	AR	STIL	SCL/TAL1 interrupting locus	Centrosome	Centriole biogenesis, spindle assembly	[42,43]
**MCPH8**	4q12	AR	CEP135	Centrosomal protein 135	Centrosome	Centriole biogenesis	[44,45]
**MCPH9**	15q21.1	AR	CEP152	Centrosomal protein 152	Centrosome	Centriole biogenesis	[39,46]
**MCPH10**	20q13.12	AR	ZNF335	Zinc finger protein 335	Nucleus	Transcription, chromatin remodeling	[47]
**MCPH11**	12p13.31	AR	PHC1	Polyhomeotic-like 1	Nucleus	Transcription, chromatin remodeling	[48]
**MCPH12**	7q21.2	AR	CDK6	Cyclin-dependent kinase 6	Cytosol/ Nucleus/ Centrosome/ Spindle poles	Cell cycle	[49]
**MCPH13**	4q24	AR	CENPE	Centromere protein E	Kinetochore	Kinetochore attachment, spindle checkpoint	[50]
**MCPH14**	1p21.2	AR	SASS6 (SAS6)	Spindle assembly abnormal protein 6 homolog	Centrosome	Centriole biogenesis	[45,51]
**MCPH15**	1p34.2	AR	MFSD2A	Major facilitator superfamily domain-containing 2A	Plasma membrane	Metabolism, cell cycle	[52,53]
**MCPH16**	12q24.33	AR	ANKLE2 (LEM4)	Ankyrin repeat and LEM domain-containing protein 2	Endoplasmic reticulum/ Nucleus	Nuclear envelope assembly	[54]
**MCPH17**	12q24.23	AR	CIT	Citron rho-interacting serine/threonine kinase	Spindle/ Midbody	Spindle assembly, cytokinesis	[55,56]
**MCPH18**	4q21.23	AD	ALFY (WDFY3)	Autophagy-linked FYVE protein	Cytoplasm/ Nucleus	Wnt signaling	[57]
**MCPH19**	3q23	AR	COPB2	Coatomer protein complex subunit Beta 2	Non-clathrin vesicles	Vesicle trafficking, apoptosis	[58]
**MCPH20**	1q32.1	AR	KIF14	Kinesin family member 14	Spindle poles/ Midbody	Spindle assembly, cytokinesis	[59,60]
**MCPH21**	12p13.31	AR	NCAPD2 (CNAP1)	Non-SMC condensin I complex subunit D2/Centrosomal Nek2-associated protein 1	Nucleus	Chromatin condensation	[61]
**MCPH22**	11q25	AR	NCAPD3	Non-SMC condensin II complex subunit D3	Nucleus	Chromatin condensation	[61]
**MCPH23**	2q11.2	AR	NCAPH	Non-SMC condensin I complex subunit H	Nucleus	Chromatin condensation	[61]
**MCPH24**	12q23.2	AR	NUP37	Nucleoporin 37	Nucleus/ Kinetochore	Nuclear pore assembly, spindle assembly	[62]
**MCPH25**	7q22.1	AR	MAP11 (TRAPPC14)	Microtubule-associated protein 11	Spindle/ Midbody/ Golgi	Spindle assembly, cytokinesis, Golgi trafficking	[63]
**MCPH26**	5q23.2	AD	LMNB1	Lamin B1	Nucleus/ Spindle	Nuclear envelope assembly, spindle assembly	[64]
**MCPH27**	19p13.3	AD	LMNB2	Lamin B2	Nucleus/ Spindle	Nuclear envelope assembly, spindle assembly	[64]
**MCPH28**	22q13	AR	RRP7A	Ribosomal RNA processing 7 homolog A	Nucleolus	Ribosome biogenesis, primary cilia resorption	[65]
**MCPH29**	9q32	AR	AKNA	AT-Hook Transcription Factor	Centrosome	Microtubule organization	[66]

AR, autosomal recessive; AD, autosomal dominant.

**Table 2:** Summary of MCPH1 mouse models.

Mouse Model	Mutation	Phenotypes							Reference
		Microcephaly	Smaller Body	Gonad Atrophy	Infertility	Tumors	DDR Defects	PCC	
***Mcph1^−/−^***	Exon 2 deletion	No	Yes	Yes	Yes	No	Yes	NR	[105]
***Mcph1^gt/gt^***	BRCT3 deletion	No	No	No	No	No	No	Yes	[111]
***Mcph1-del***	Exon 4-5 deletion	Yes	Yes	Yes	Yes	Yes	Yes	Yes	[81,120]
***Mcph1^tm1a/tm1a^***	Exon 4 deletion	Yes	No	NR	Yes	NR	Yes	NR	[122]
***Mcph1-ΔBR1***	BRCT1 deletion	Yes	Yes	Yes	Yes	Yes	Yes	Yes	[91]

Note: DDR, DNA damage response; PCC, premature chromosome condensation; NR: not reported.
